# Managing heart failure with reduced ejection fraction merged with myocardial infarction with non-obstructive coronary arteries: a case report

**DOI:** 10.1093/ehjcr/ytae540

**Published:** 2024-09-28

**Authors:** So Ikebe, Masahiro Yamamoto, Masanobu Ishii, Eiichiro Yamamoto, Kenichi Tsujita

**Affiliations:** Department of Cardiovascular Medicine, Graduate School of Medical Sciences, Kumamoto University Hospital, 1-1-1 Honjo, Chuo-ku, Kumamoto 860-8556, Japan; Department of Cardiovascular Medicine, Graduate School of Medical Sciences, Kumamoto University Hospital, 1-1-1 Honjo, Chuo-ku, Kumamoto 860-8556, Japan; Department of Cardiovascular Medicine, Graduate School of Medical Sciences, Kumamoto University Hospital, 1-1-1 Honjo, Chuo-ku, Kumamoto 860-8556, Japan; Department of Medical Information Science, Graduate School of Medical Sciences, Kumamoto University Hospital, 1-1-1 Honjo, Chuo-ku, Kumamoto 860-8556, Japan; Department of Cardiovascular Medicine, Graduate School of Medical Sciences, Kumamoto University Hospital, 1-1-1 Honjo, Chuo-ku, Kumamoto 860-8556, Japan; Department of Cardiovascular Medicine, Graduate School of Medical Sciences, Kumamoto University Hospital, 1-1-1 Honjo, Chuo-ku, Kumamoto 860-8556, Japan; Center of Metabolic Regulation of Healthy Aging, Faculty of Life Sciences, Kumamoto University Hospital, 1-1-1 Honjo, Chuo-ku, Kumamoto, Japan

**Keywords:** MINOCA, Heart failure, Ejection fraction, Functional coronary angiography, VSA, CSA

## Abstract

**Background:**

The concepts of myocardial infarction with non-obstructive coronary arteries (MINOCA) are now widely accepted. Calcium channel blockers (CCBs) are the first-line medication for coronary spastic angina (coronary spastic angina: CSA/vasospastic angina: VSA), while β-blockers sometimes do not improve CSA/VSA. However, β-blockers are essential for managing symptoms of coronary microvascular dysfunction and considered vital for treating heart failure with reduced ejection fraction (HFrEF).

**Case summary:**

We present the case of an 83-year-old female admitted with shortness of breath persisting for over 1 year and worsening ejection fraction (EF) from 65% to 32%. On admission, she experienced chest pain at rest despite finding no significant stenosis on coronary angiography. Several days later, we performed functional coronary angiography (FCA), revealing diffuse epicardial coronary spasm upon injecting acetylcholine. The coronary flow reserve was 4.4 (≧2.0), and the microvascular resistance index was 20 (<25). We diagnosed the patient with a myocardial injury event induced by CSA/VSA and initiated dihydropyridine CCBs. A few months later, her chest pain resolved; the HF symptoms improved (NYHA: from Ⅲ to Ⅱ), accompanied by a reduction in B-type natriuretic peptide levels (from 4561.2 to 75.4 pg/mL) and EF improvement (from 32.0% to 62.6%).

**Discussion:**

We managed a patient with HFrEF and MINOCA. Although CCBs are not routinely recommended for HFrEF, we added dihydropyridine CCBs to treat CSA/VSA based on comprehensive diagnostic procedures. This approach sedated chest pain and may have contributed to her EF improvement. Detailed examinations and tailored treatment strategies might be helpful for HF treatment.

**Learning points**

- The patient experienced episodes of chest pain at rest and elevated myocardial enzymes, and functional coronary angiography (FCA) identified myocardial infarction with non-obstructive coronary arteries (MINOCA).
- We initiated dihydropyridine CCBs, and a few months later, her chest pain resolved, and heart failure symptoms improved. We diagnosed this case as heart failure with reduced ejection fraction (HFrEF) merged with epicardial coronary vasospasm.

## Introduction

Myocardial infarction with non-obstructive coronary arteries (MINOCA) and ischemic non-obstructive coronary artery disease (INOCA) are often misdiagnosed and overlooked because they involve non-obstructed coronary arteries. Emerging diagnostic, risk stratification, and personalized treatment strategies could address this issue.

Treatment strategies for heart failure with reduced ejection fraction (HFrEF) have advanced, focusing on the combination of multiple drugs. Guideline-directed medical therapy (GDMT) is essential and recommends that patients with HFrEF should be treated with a combination of the four drugs, including β-blockers, to achieve substantial and lasting reductions in mortality, heart failure hospitalizations, and symptoms.^1^ However, administering calcium channel blockers (CCBs) to patients with HFrEF is generally avoided due to concerns about worsening heart failure.^2^ CCBs are the first-line medication for epicardial coronary spastic angina (coronary spastic angina: CSA/vasospastic angina: VSA).^3^ Clinically, distinguishing between chest pain caused by heart failure, coronary angina, and momentary infarction events can be challenging. Additionally, limited evidence and reports on treatment strategies for HFrEF combined with MINOCA exist. We report the case of an 83-year-old female who was managed for HFrEF with MINOCA using CCBs.

## Summary figure

**Figure:**
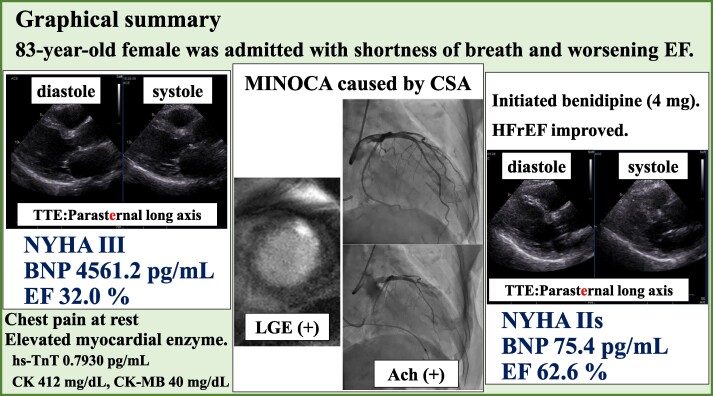


## Case presentation

The patient, an 83-year-old female, presented with shortness of breath (SOB). At 81 years old, there were no notable abnormalities; left ventricular (LV) function was normal [ejection fraction (EF): 65%]. By 82 years old, she experienced worsening SOB and decreased LV contraction (EF: 47%). Despite treatment with sacubitril/valsartan (200 mg/day), her symptoms persisted, and EF decreased further [New York Heart Association (NYHA): class Ⅲ, EF: 32%]. Consequently, she was admitted for thorough examination and treatment. Her vital signs and medications upon admission are presented in *Table 1*.

**Table 1:** Vital signs, medications, and laboratory data on admission and discharge

Vital signs	Admission	Discharge
Height	142.3 cm	
Body weight	46.6 kg	43.1 kg
Blood pressure (systolic/diastolic)	111/93 mmHg	99/71 mmHg
Pulse rate	61 bpm	58 bpm
SpO2	97% (on room air)	96% (on room air)
Course crackles	(+)	(−)
Wheezes	(+)	(−)
Pitting oedema	(+)	(−)
**Medications**		
Sacubitril/valsartan	200 mg	200 mg
Carvedilol		2.5 mg
Spironolactone		12.5 mg
Dapagliflozin		10 mg
Benidipine		4 mg
Furosemide	10 mg	20 mg
Raloxifene	60 mg	
Teprenone	50 mg	
Rabeprazole	10 mg	

Bpm, beat per minute; SpO2, oxygen saturation.

**Figure 1:**
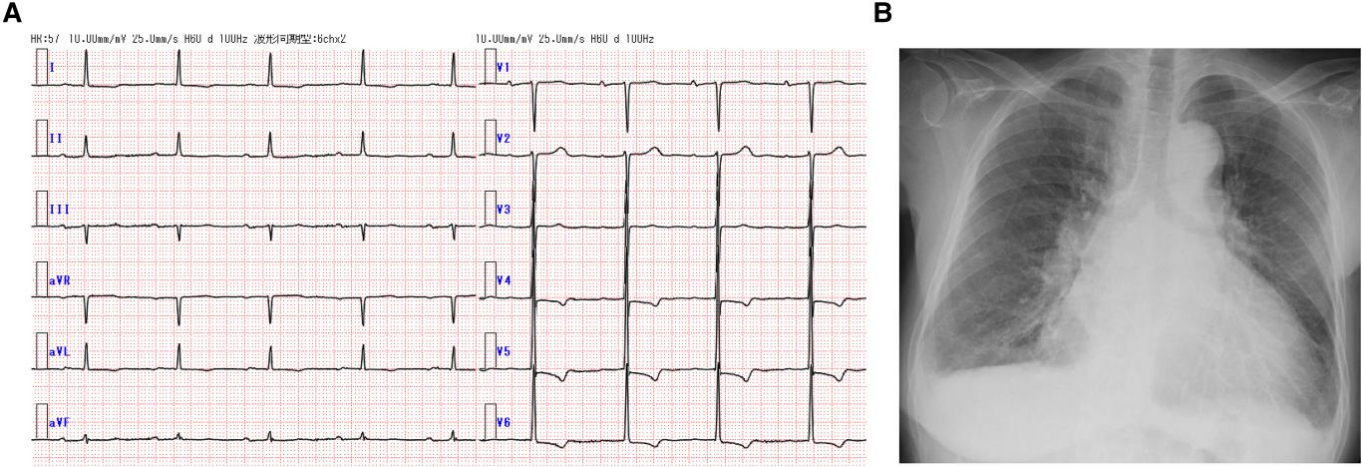
(*A*) Twelve-lead electrocardiogram shows sinus rhythm and T wave inversion in leads I, aVL, and V4-6. (*B*) Chest X-ray showed enlargement of the cardiac silhouette, and costophrenic angles are dull.

Upon auscultation, we heard coarse crackles, observing pitting oedema on both lower limbs. The B-type natriuretic peptide (BNP) level was notably elevated at 4561.2 pg/mL. Electrocardiography showed sinus rhythm with T wave inversion in leads I, aVL, and V4–6 (*Figure 1A*). Chest X-ray showed cardiac silhouette enlargement with dull costophrenic angles (*Figure 1B*). Her echocardiogram findings are shown in Supplementary material online, *Video S1A* and *B*. Transthoracic echocardiogram (TTE) showed reduced EF (32.0%) with diffuse severe hypokinesis and left ventricular diastolic dimension (LVDd) and left ventricular systolic dimension (LVDs) measured as 58 and 40 mm, respectively.

**Figure 2:**
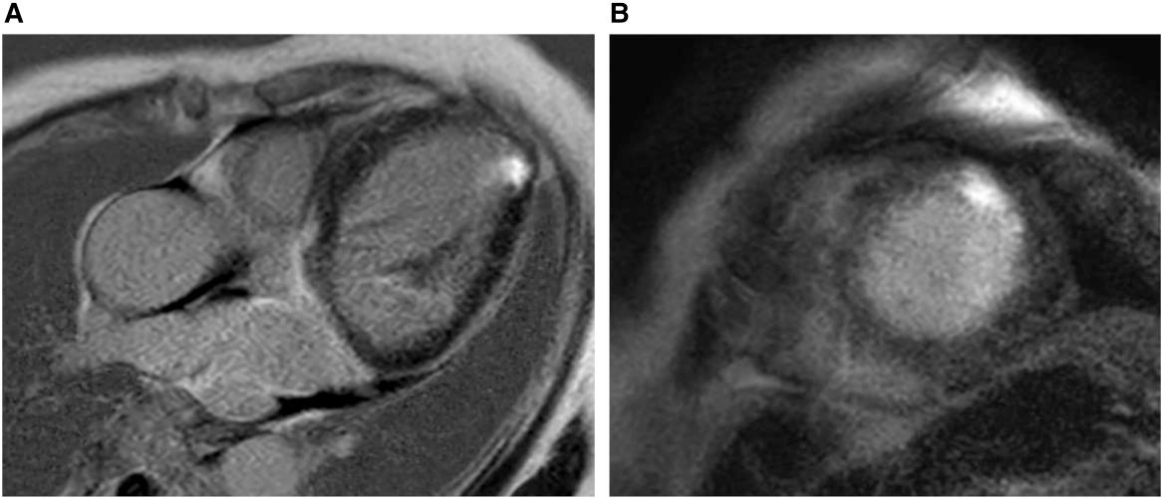
CMRI reveals LGE at the endocardial side of the left ventricle's anterior wall about (*A*) long axis view and (*B*) short axis view. CMRI, gadolinium-enhanced cardiovascular magnetic resonance imaging; LGE, late gadolinium enhancement.

**Figure 3:**
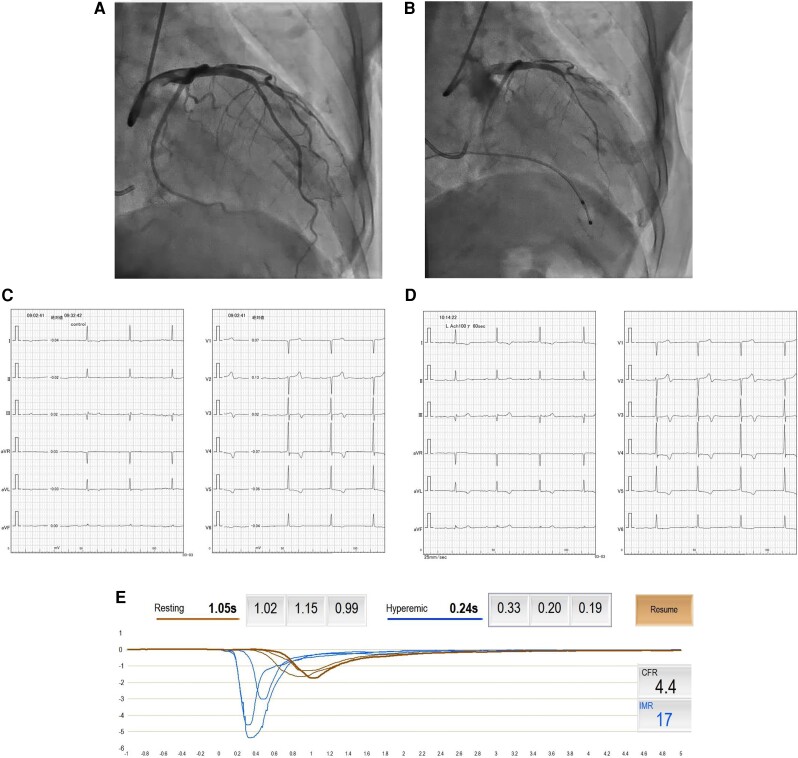
ICA and electrocardiography during coronary reactivity testing. (*A*) Baseline angiogram demonstrating no ischemic abnormalities. (*B*) After 100 μg acetylcholine injection induced diffuse epicardial spasm. (*C*), (*D*) Twelve-lead electrocardiogram shows T-wave of Ⅱ, Ⅲ, and aVF leads got horizontalized with her chest pain. (*E*) CFR/IMR thermodilution curves. The numbers in the figure show the mean transit time. Blood pressure of the point of transducer is 86 mmHg. ICA, Invasive coronary angiography.

**Figure 4:**
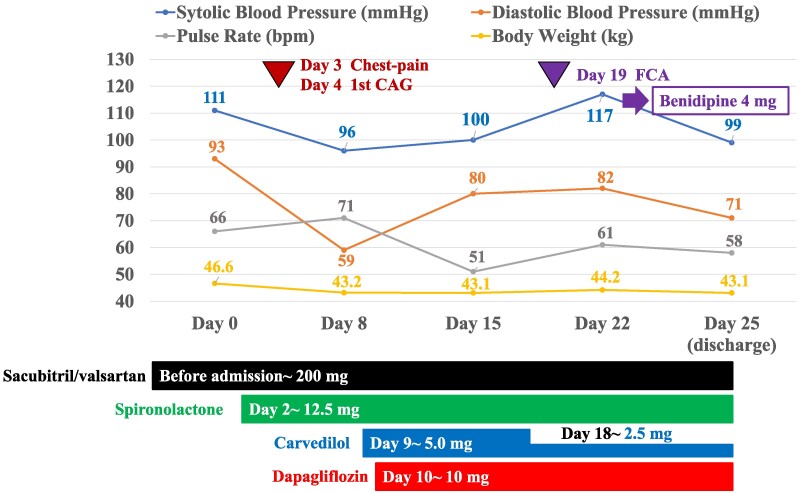
Vital signs and additional medications during hospitalization.

We increased the dose of furosemide and added carvedilol (2.5 mg) and spironolactone (25 mg). Within 2 days, her body weight (BW) reduced by 1 kg (BW:45.6 kg), and her SOB improved. However, on the third day, she experienced sudden chest pain at rest, which lasted a few minutes, and then subsided. The following day, her electrocardiogram showed new T-wave inversion in leads V2–3 (see Supplementary material online, *Figure S1*). Additionally, myocardial enzymes were elevated (peak hs-TnT: 0.7930 pg/mL, peak CK: 412 mg/dL, peak CK-MB: 40 mg/dL). Coronary angiography (CAG) showed no significant stenosis (see Supplementary material online, *Video S2A* and *B*). Her history of chest pains revealed that she had experienced chest pains at rest several times per month over the past year. Additionally, gadolinium-enhanced cardiovascular magnetic resonance imaging (CMRI) revealed late gadolinium enhancement (LGE) on the endocardial side of the left ventricle’s anterior wall (*Figure 2A* and *B*). We performed functional coronary angiography (FCA). Following the injection of 100 μg acetylcholine, we observed diffuse epicardial spasm of the left anterior descending coronary artery (LAD) (*Figure 3A* and *B* and Supplementary material online, *Video S3A* and *B*). On the electrocardiogram, T-waves in leads Ⅱ, Ⅲ, and aVF flattened during her chest pain (*Figure 3C* and *D*). After remission of spasm by nitroglycerin injection, we performed coronary flow reserve (CFR) and index of microvascular resistance (IMR). The coronary flow reserve was 4.4 (normal: ≧2.0), and the index of microvascular resistance was 20 (normal <25) (*Figure 3E*). In addition, myocardium biopsy was also performed. Pathological examination showed only nonspecific myocardial fibrosis; we excluded other secondary cardiomyopathies. Based on these findings, we diagnosed the patient with CSA/VSA and initiated benidipine (4 mg), which is dihydropyridine CCBs (*Figure 4*).

Following treatment, the patient experienced no further chest pain; symptoms of congestive heart failure improved. She was discharged from the hospital with compensated heart failure (NYHA Ⅱ, BW 43.5 kg). Three months later, echocardiogram (see Supplementary material online, *Video S4*) showed improved LV contraction. Compared with admission, her EF improved from 32.0% to 63.6%, and the LV chamber size decreased (LVDd: 58.5 to 46.6 mm, LVDs: 40.1 to 26.9 mm), with LV contraction changing from diffuse severe hypokinesis to mild hypokinesis at the apex. BNP levels decreased from 4561.2 to 75.4 pg/mL. Her vital signs and medications upon discharge are summarized in *Table 1*. She has continued to attend our clinic since then and has not had any recurrence of chest pain. Finally, the patient was diagnosed with HFrEF, decompensated heart failure, and MINOCA.

## Discussion

The noteworthy points of this case are as follows: first, despite administering first-line drugs for heart failure with reduced ejection fraction (HFrEF), the patient's heart failure worsened; second, elevated biomarkers and CMRI findings showed myocardial injury; third, CAG revealed no significant fixed stenosis in the epicardial coronary arteries; and fourth, FCA confirmed CSA/VSA.

To the best of our knowledge, there is limited detailed evidence regarding HFrEF combined with MINOCA or INOCA. Coronary arteries consist of two main groups: epicardial coronary arteries and coronary micro-vessels, both of which can exhibit structural and functional abnormalities.^4^ According to the universal definition, MI is characterized by acute myocardial injury detected via myocardial biomarkers due to acute myocardial ischaemia.^5^ MINOCA is diagnosed when there is evidence of MI without occlusive epicardial coronary artery lesions.^6^ Diagnosis of MINOCA typically involves CMRI and FCA.^2^ In our case, the patient experienced episodes of chest pain at rest and elevated myocardial enzymes. CMRI revealed LGE on the endocardial side of the left ventricle’s anterior wall, suggesting a myocardial event in the region supplied by the LAD coronary artery. The major causes of myocardial ischemia in the absence of obstructive coronary artery disease include CSA/VSA and coronary microvascular dysfunction (CMD), which manifest as vasospastic angina and microvascular angina. FCA is needed to identify these abnormalities. Generally, major adverse cardiac events and HF re-hospitalization risk are lower in patients with MINOCA compared with those with MI with obstructive coronary artery disease (MI-CAD).^7^ However, 5.9% of older patients (>65 years old) with MINOCA experienced HF re-hospitalization within 12 months.^7^ Recent studies have suggested that reduced EF (<50%) is an independent predictor for HF re-hospitalization in patients with MINOCA;^8^ LGE has been identified as an independent predictor of adverse cardiac events.^9^ These parameters can be considered high-risk markers in patients with MINOCA.

In clinical practice, including our case, differentiating between chest pain caused by heart failure and other factors can be challenging. Additionally, β-blockers, a key drug for HFrEF, are useless in CSA/VSA. Therefore, achieving the target dose of GDMT, including up-titration, is crucial for achieving prognostic and clinical effects for HFrEF.^10,11^ Properly diagnosing coronary spasms in patients with angina at rest is important for formulating appropriate treatment strategies.

β-Blockers are effective in treating exertional angina associated with CMD.^4^ FCA also offers insights into whether β-blockers, which can induce coronary spasms, should be prescribed. CCBs are not recommended for HFrEF patients without angina.^12^ However, CCBs are the first-line treatment of epicardial coronary vasospasm.^2^ We decided to add CCBs as treatment of MINOCA caused by CSA/VSA, which was diagnosed by comprehensive diagnosing process. Additionally, we selected dihydropyridine CCBs to avoid heart failure worsening.^13^ Given the patient's history of chest pain preceding heart failure progression and the results of various examinations, we diagnosed this case with HFrEF aggravated by epicardial coronary vasospasm. Subsequently, the HF improved.

This case revealed a notable response to dihydropyridine CCB pharmacological therapy, suggesting its efficacy in managing the condition. Recognizing that MINOCA or INOCA could contribute to HFrEF is important. Therefore, detailed examinations and tailored treatment strategies can benefit HFrEF treatment.

## Lead author biography


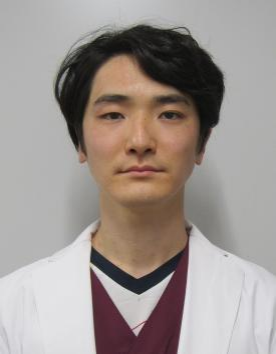


So Ikebe is a doctor working in Kumamoto University, Kumamoto, Japan. He performed a fellowship in Kumamoto University, Kumamoto, Japan. His areas of interest include heart failure, ischaemic heart disease, and intervention.

## Supplementary Material

ytae540_Supplementary_Data

## Data Availability

The data underlying this article are available in the article and in its online Supplementary material.
