# Supplementary figures and images for: Managing heart failure with reduced ejection fraction merged with myocardial infarction with non-obstructive coronary arteries: a case report
Source: Eur Heart J Case Rep. 2024 Sep 28;8(10):ytae540. doi: 10.1093/ehjcr/ytae540 (PMC11500752; doi:10.1093/ehjcr/ytae540)

**Supplemental Figure 1.**

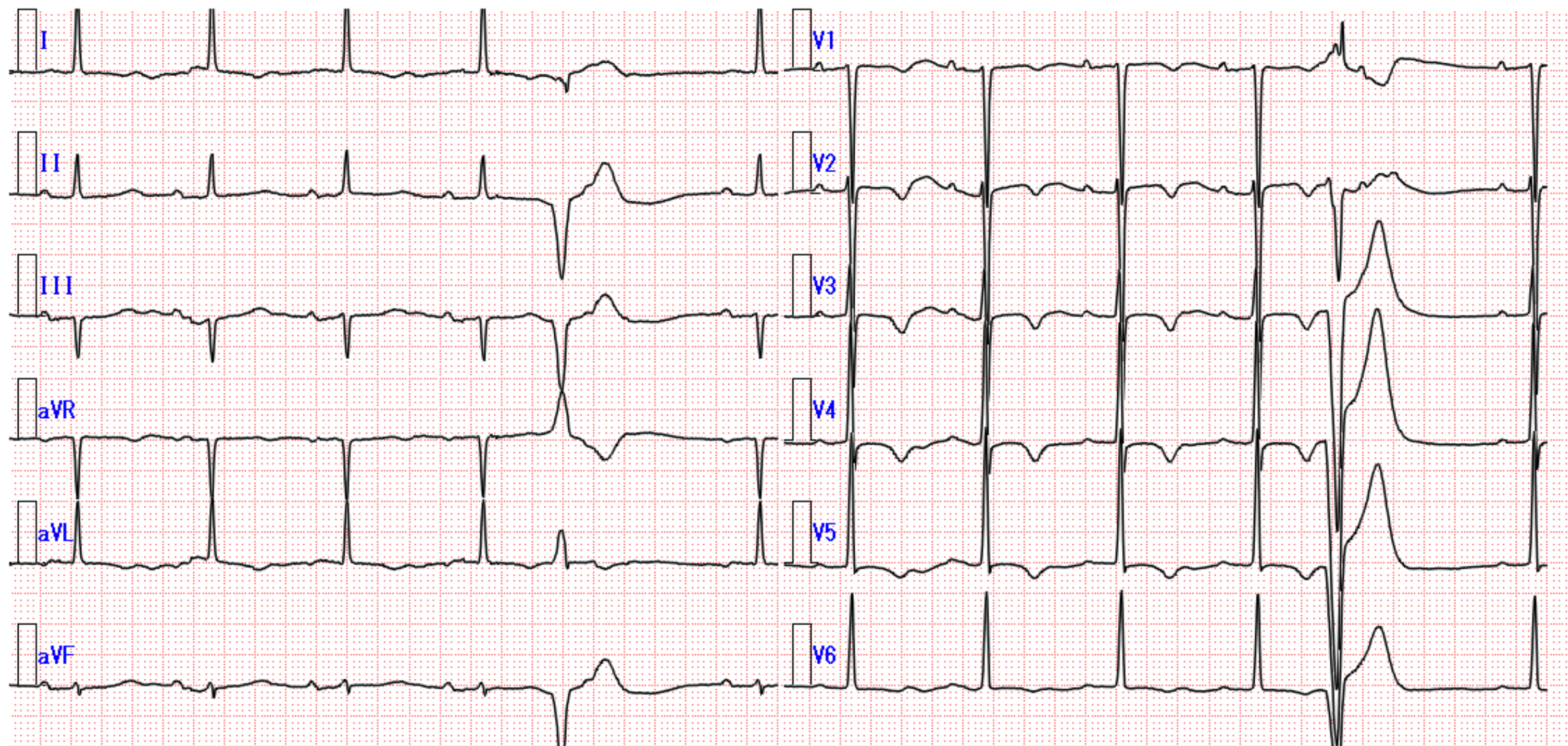

Supplement: ytae540_Supplementary_Data [file ytae540_supplementary_data.zip › Supplemental Figure 1.pdf]

## Slide 1
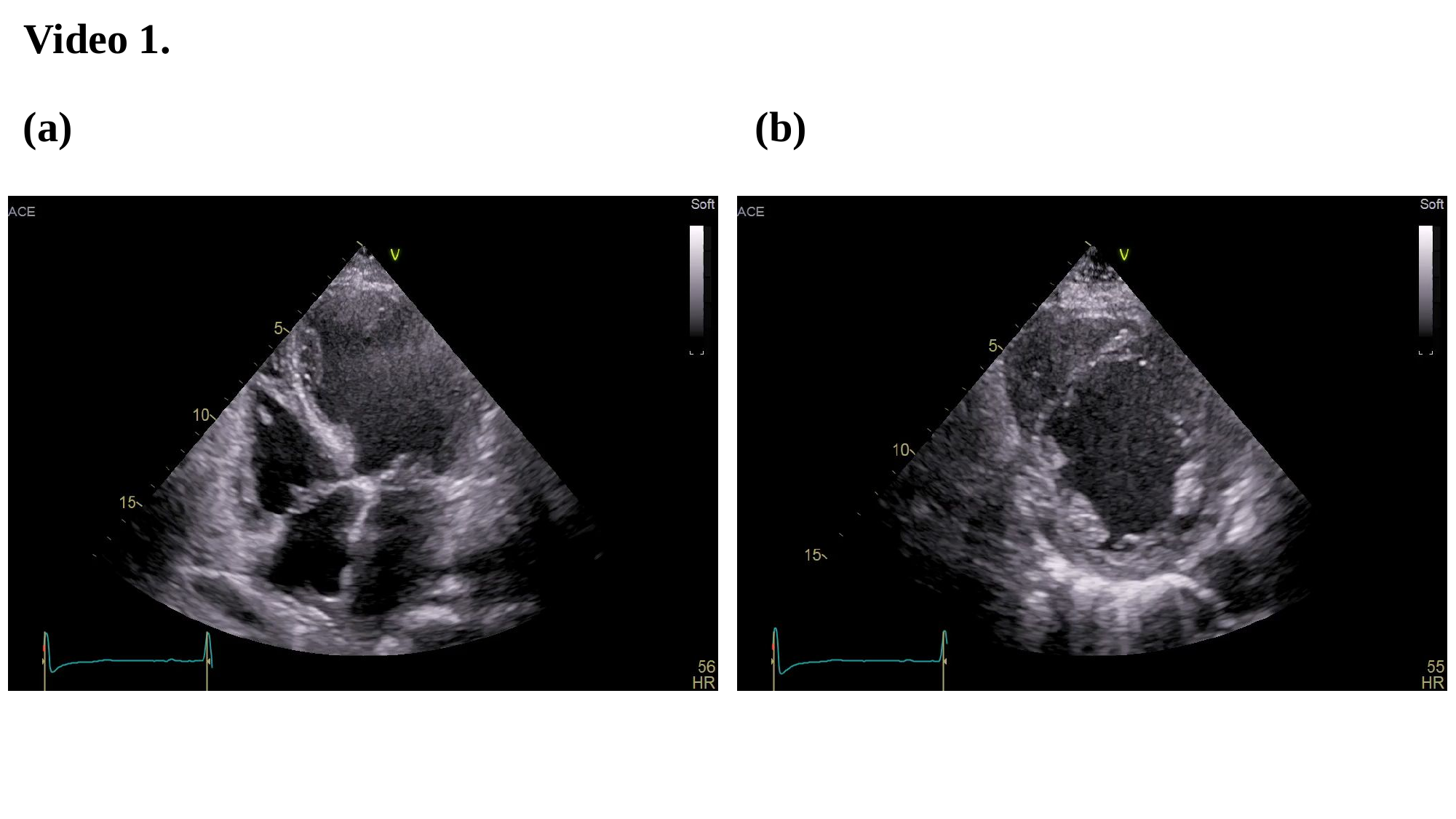

Video 1.
(a)
(b)

Supplement: ytae540_Supplementary_Data [file ytae540_supplementary_data.zip › Video 1.pptx]

## Slide 1
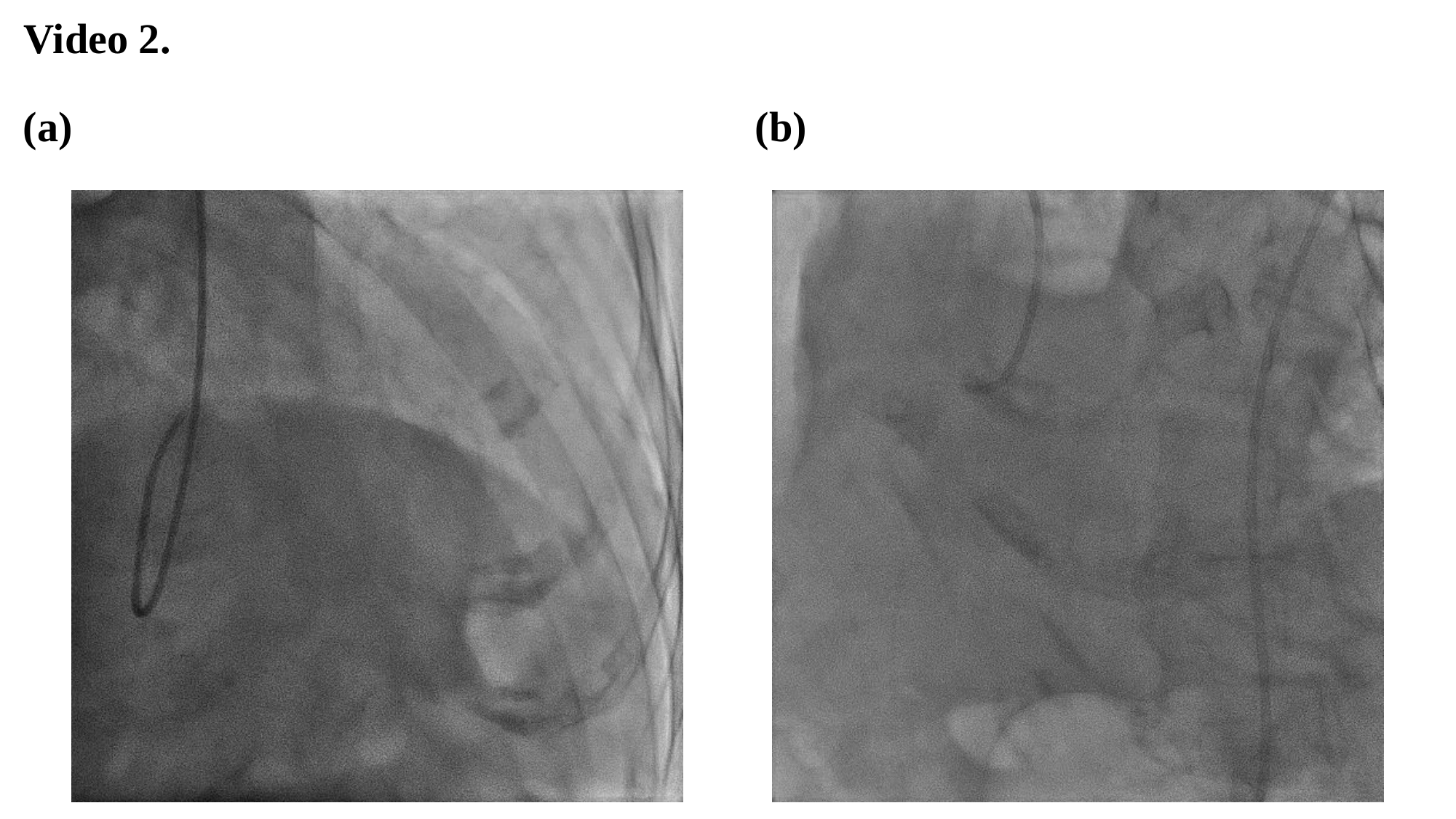

Video 2.
(a)
(b)

Supplement: ytae540_Supplementary_Data [file ytae540_supplementary_data.zip › Video 2.pptx]

## Slide 1
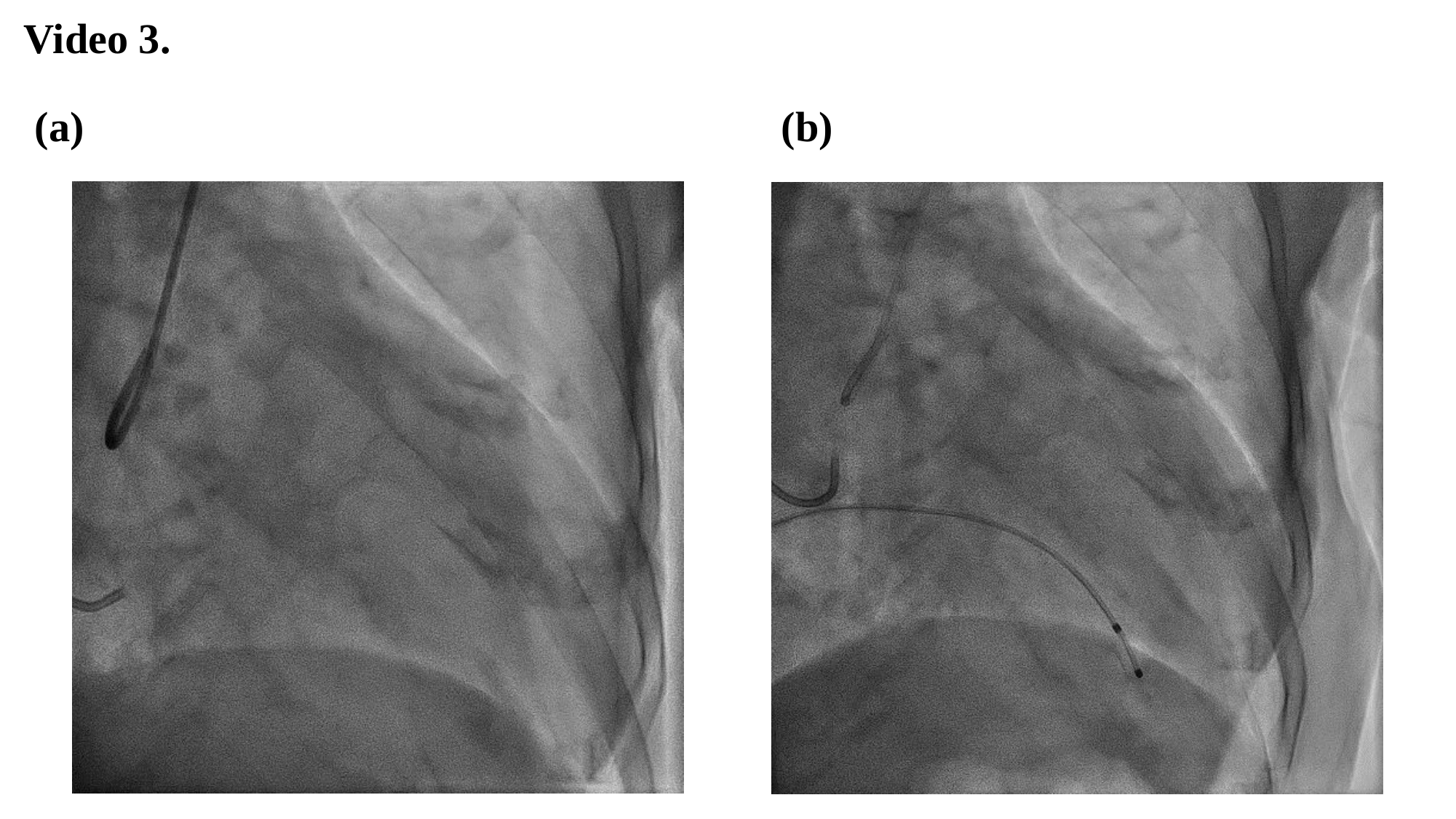

Video 3.
(a)
(b)

Supplement: ytae540_Supplementary_Data [file ytae540_supplementary_data.zip › Video 3.pptx]

## Slide 1
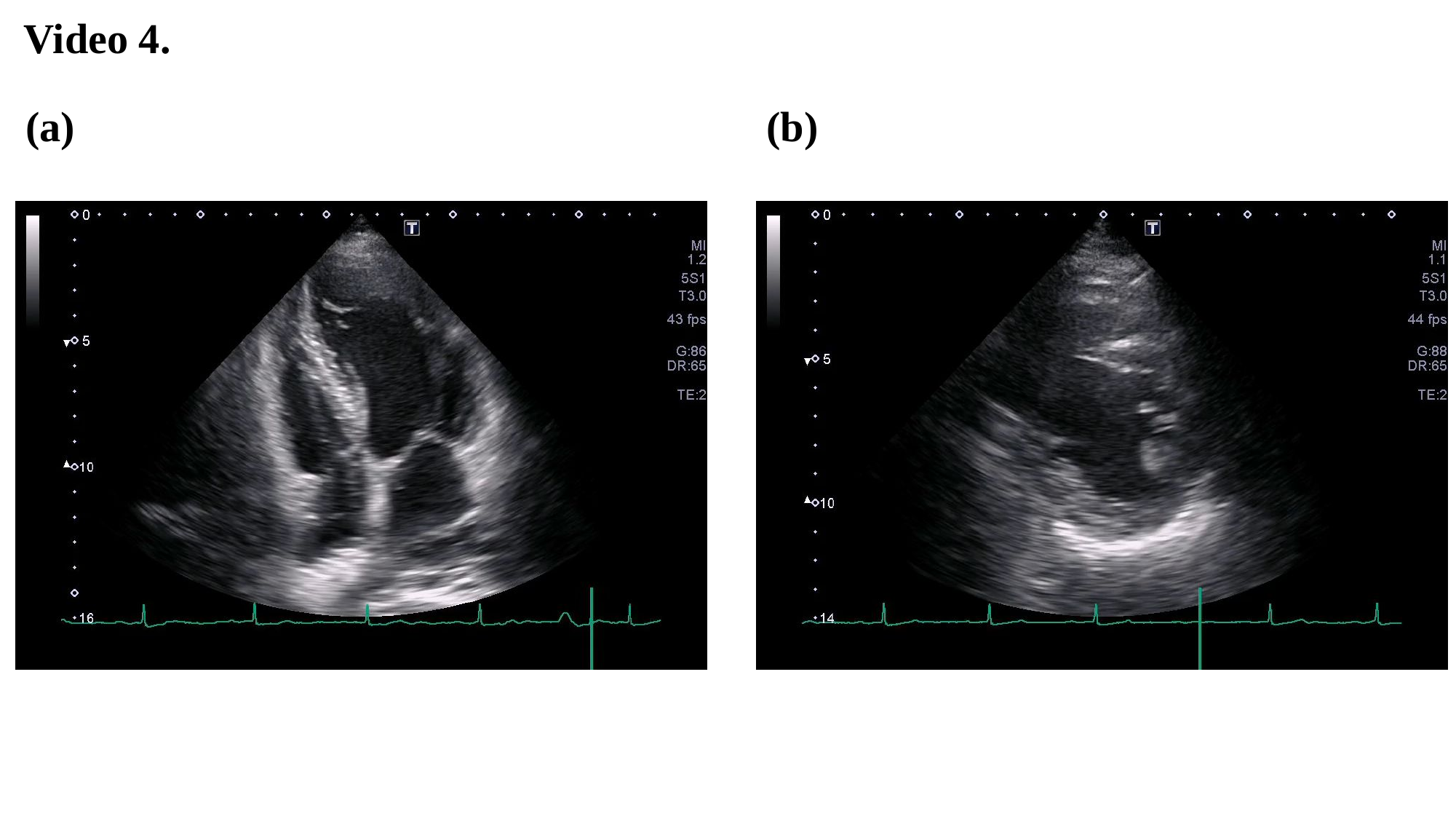

Video 4.
(a)
(b)

Supplement: ytae540_Supplementary_Data [file ytae540_supplementary_data.zip › Video 4.pptx]
